# Osteopontin in relation to Prognosis following Coronary Artery Bypass Graft Surgery

**DOI:** 10.1155/2016/1868739

**Published:** 2016-08-14

**Authors:** Eftihia Sbarouni, Panagiota Georgiadou, Sofia Chatzikyriakou, Antonis Analitis, Antigoni Chaidaroglou, Demitris Degiannis, Vassilis Voudris

**Affiliations:** ^1^2nd Division of Interventional Cardiology, Onassis Cardiac Surgery Center, 17674 Athens, Greece; ^2^Department of Hygiene, Epidemiology and Medical Statistics, Medical School, University of Athens, 11527 Athens, Greece; ^3^Molecular Immunopathology and Histocompatibility Laboratory, Onassis Cardiac Surgery Center, 17674 Athens, Greece

## Abstract

Cardiovascular events may occur even after complete revascularization in patients with coronary artery disease. We measured preoperative osteopontin (OPN) levels in 131 consecutive patients (66.5 ± 10 years old, 117 men and 14 women) with left ventricular ejection fraction of 50.7 ± 9.2% and low logistic EuroScore (3.5 ± 3.2%) undergoing elective Coronary Artery Bypass Grafting (CABG) surgery. Patients were prospectively followed up for a median of 12 months (range 11–24). The primary study endpoint was the composite of cardiovascular death, nonfatal myocardial infarction, need for repeat revascularization, and hospitalization for cardiovascular events. Pre-op OPN plasma levels were 77.9 (49.5, 150.9). Patients with prior acute myocardial infarction (AMI) had significantly higher OPN levels compared to those without [131.5 (52.2, 219) versus 73.3 (45.1, 125), *p* = 0.007]. OPN levels were positively related to EuroScore (*r* = 0.2, *p* = 0.031). Pre-op OPN levels did not differ between patients who had a major adverse event during follow-up compared to those with no event (*p* = 0.209) and had no effect on the hazard of future adverse cardiac events [HR (95% CI): 1.48 (0.43–4.99), *p* = 0.527]. The history of AMI was associated with increased risk of subsequent cardiovascular events at follow-up (*p* = 0.02). OPN is associated with preoperative risk assessment prior to low-risk CABG but did not independently predict outcome.

## 1. Introduction

Osteopontin (OPN) is a matrix cellular protein and cytokine, released when tissue injury and remodeling occur in various organs including the heart [1]. OPN has been isolated in human aorta, carotid, and coronary arteries and has been shown to be involved in the development and progression of atherosclerosis [2]. OPN may have opposite effects during several cardiovascular diseases [1]. OPN seems to affect prognosis in coronary artery disease (CAD), including acute coronary syndromes (ACS) and chronic stable angina [3, 4], and in heart failure [5].

Cardiovascular events may occur even after complete revascularization and there is constant interest in independent markers of subsequent risk including biomarkers [6]. We sought to investigate whether OPN pre-op has prognostic value for patients following elective Coronary Artery Bypass Grafting (CABG) surgery.

## 2. Materials and Methods

### 2.1. Study Population

We studied 131 consecutive patients undergoing elective CABG in our institution. Patients with acute or chronic inflammatory disease, immunological disease, any cancer, and administration of any vitamin supplement during the last 6 months were excluded from the study. All patients gave informed consent and the study protocol was approved by the Ethics Committee of our institution.

There were 117 men and 14 women with a mean age of 66.5 ± 10 years (Table 1). Demographic characteristics in relation to risk factors for CAD are shown in Table 2. Six (4.7%) of the diabetics were treated with insulin. Regarding previous acute myocardial infarction (AMI) and revascularization procedures, 42 (32.6%) had a history of AMI and 31 (23.8%) had undergone percutaneous coronary intervention (PCI) and 5 (3.9%) CABG. Almost a fourth, 29 (22.3%), suffered an ACS preoperatively. The majority of our patients presented with significant left main or 3-vessel disease, 98 (76%), and only 29 (22.5%) and 2 (1.6%) were operated on for 2- or 1-vessel disease, respectively. The study sample was relatively at low risk with preserved ejection fraction (50.7 ± 9.2%) and low logistic EuroSCORE (European system for cardiac operative risk evaluation) (3.5 ± 3.2%).

### 2.2. Biochemical Analysis

Preoperative blood samples were obtained for OPN evaluation. All samples were centrifuged for 10 min in 3000 rounds per min within 15 min of sampling and stored at −80°C until assayed. Plasma OPN levels were determined by an enzyme-linked immunosorbent assay (ELISA) using the Quantikine human OPN kit (R&D Systems, UK) and are expressed in ng/mL. The average of duplicate readings for each standard, control, and sample was used. The standard curve was created by using a four-parameter logistic curve fit. Samples were usually diluted 25 times. The mean minimum detectable dose of OPN was 0.011 ng/mL. The intra- and interassay coefficients of variations were 4% and 7%, respectively.

### 2.3. Patient Follow-Up

All patients but one were operated on on-pump. The number of grafts inserted and the cross-clumping and cardiopulmonary bypass time are shown in Table 1. Median hospital stay was 8 days (range 3–28). There was only one in-hospital death. Two patients developed acute renal failure and improved on dialysis and one suffered tamponade which was drained successfully. Seven patients had chronic atrial fibrillation; of those with sinus rhythm, 44 patients developed atrial fibrillation postoperatively, which was successfully cardioverted with intravenous amiodarone.

All patients were prospectively followed up for a median of 12 months (m) (range 11–24). The primary study endpoint was the composite of cardiovascular death, nonfatal myocardial infarction, need for repeat revascularization, and hospitalization for cardiovascular events. Nine patients reached the primary endpoint during follow-up. There was one sudden cardiac death 9 m post-op. One patient suffered a nonfatal non-ST elevation myocardial infarction, 18 m post-op. We recorded 4 cases of repeat revascularization procedures: one PCI to circumflex artery 1 m post-op due to ACS and incomplete revascularization of the dominant circumflex, one PCI to the ungrafted right coronary artery which was disease-free at the time of the operation 11 m post-op and PCI to the left descending anterior artery distal to the internal mammary artery anastomosis, both at the same stage, and two PCIs to the intermediate and the first obtuse marginal, respectively, both new lesions in relation to the pre-op angiogram. Hospitalization occurred as follows: one patient was operated on for peripheral arterial disease with aortofemoral bypass 9 m post-op, another patient developed an embolic event in his right leg 2 m post-op, which was treated percutaneously, and one patient underwent coronary angiography for unstable angina and was subsequently managed medically. In addition, 3 patients were hospitalized for respiratory infections and 4 for minor surgery, 4 were operated on for cancer, and 1 was admitted for renal failure which improved with medical therapy and 1 patient for worsening heart failure, who was managed conservatively. Paroxysmal atrial fibrillation occurred in 3 patients; all of them had atrial fibrillation immediately postoperatively.

### 2.4. Statistical Analysis

Association between categorical variables was tested using chi-square or Fisher's exact test. Spearman correlation was applied to investigate associations between OPN and continuous variables. Cox proportional hazard models were used to estimate the potential effect of OPN on the hazard of major cardiovascular event. All tests were two-sided at *a* = 5% level of statistical significance.

## 3. Results

**Figure 1:**
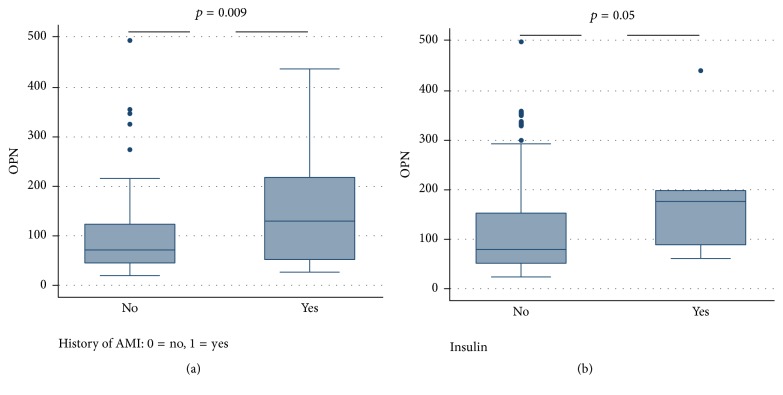
(a) Significant differences in osteopontin levels between patients with and without history of acute myocardial infarction. (b) Significant differences in osteopontin levels between patients treated with insulin compared to those not on insulin (box plots showing median, interquartile range).

Pre-op OPN plasma levels were 77.9 (49.5, 150.9) [median (interquartile range 25th–75th percentiles)]. There was no difference in OPN levels in relation to age or gender and the prevalence of the examined cardiovascular risk factors (hypertension, diabetes, hyperlipidemia, smoking, and family history of coronary disease) (Table 2). Interestingly, however, patients with prior AMI had significantly higher OPN levels compared to those without [131.5 (52.2, 219) versus 73.3 (45.1, 125), *p* = 0.007] (Figure 1(a)). Likewise OPN was significantly higher in patients treated with insulin when compared to those not on insulin [170 (84.6, 196) versus 77.3 (49.5, 149), *p* = 0.05] (Figure 1(b)). OPN was lower in patients with 1- or 2-vessel disease, 65.3 (43.3, 149), compared to patients with 3-vessel disease or left main, 82.3 (53.3, 158), but this difference did not reach statistical significance (*p* = 0.23). In addition, OPN showed no relation to left ventricular ejection fraction (*r* = −0.09, *p* = 0.31). Conversely, OPN levels were positively related to EuroSCORE (*r* = 0.2, *p* = 0.031). Pre-op OPN levels did not differ between patients who had a major adverse event during follow-up compared to those who did not experience any event during this period [116 (54.9, 181.2) versus 77.4 (49.2, 149), *p* = 0.209]. Furthermore, pre-op OPN levels had no effect on the hazard of future major adverse cardiac events [hazard ratio (95% confidence interval (CI)): 1.48 (0.43–4.99), *p* = 0.527]. As expected, history of AMI was associated with increased risk of subsequent cardiovascular events at follow-up [hazard ratio (95% CI): 4.46 (1.27–15.66), *p* = 0.02], even after controlling for OPN levels.

## 4. Discussion

We evaluated pre-op OPN levels in patients with stable CAD undergoing elective CABG on-pump and found that OPN is higher in patients with prior AMI and those on insulin; OPN does not vary in relation to CAD severity or left ventricular ejection fraction but increases with higher EuroSCORES. In addition, OPN does not predict postoperative cardiovascular events but, as expected, a history of AMI does.

Plasma OPN levels may be associated with the presence and extent of CAD [7]; in multivariate analysis, however, when atherosclerotic risk factors are taken into account the severity of coronary atherosclerosis is not an independent factor for OPN levels [8]. In addition, OPN has been shown to predict risk for future cardiovascular events in patients undergoing PCI [9]; OPN, however, was higher in patients with coronary calcification and correlated with the number of calcified segments in this study [10] although it is known that cardiovascular events are often associated with the presence of soft atheromatous plaques [11]. We have previously shown that OPN levels do not differ between patients with and without CAD and we now found that OPN levels are similar in relation to the severity of CAD as expressed by the number of the diseased vessels [11].

OPN was inversely related to left ventricular ejection fraction [4] and OPN > 55 ng/mL was an independent predictor of adverse outcome. In this study, however, we did not confirm either the relation to left ventricular performance or the prognostic value. Circulating OPN seems to be positively related to left ventricular dimensions and inversely to ejection fraction mostly in postmyocardial infarction patients [12]; in our study only one-third of patients had a history of AMI and these patients had higher OPN levels and worse outcome. In addition, our short follow-up and complete surgical revascularization may have contributed to low event rate and lack of prognostic significance of OPN.

High glucose increases OPN m-RNA in smooth muscle cells [13]; there is increased OPN in diabetic arteries [14]. We did not observe different OPN levels in relation to diabetes, when we compared diabetics, irrespective of treatment, with nondiabetics but OPN was higher in patients on insulin versus those without insulin. There is evidence that insulin-dependent diabetic patients with CAD present with increased levels of cytokines independently of glycemic control, the duration of diabetes, and the extent of CAD [15].

Our results suggest a positive relationship between plasma OPN levels and the surgical risk in stable patients undergoing elective CABG. EuroSCORE point system includes preoperative risk factors, such as age, peripheral arterial disease, left ventricular ejection fraction, and thoracic aorta surgery [16], parameters which in some studies are related to OPN levels [4]. In our cohort, however, OPN did not differ in relation to age and left ventricular performance.

## 5. Conclusion

OPN is associated with preoperative risk assessment prior to low-risk patients undergoing CABG but did not independently predict outcome in the present study. Further research is required with larger number of patients to investigate if pre-op OPN could eventually replace or ameliorate the performance of EuroSCORE for the evaluation of cardiac operative mortality risk.

## Figures and Tables

**Table 1:** Baseline characteristics and data of coronary artery bypass grafting procedure in study population.

Age (yrs), mean ± SD^a^	66.5 ± 10

Height (cm), mean ± SD	171 ± 8

Weight (kg), mean ± SD	84 ± 17

Body mass index (kg/m^2^), mean ± SD	29 ± 5

Osteopontin (ng/mL) median (interquartile range 25th–75th percentiles)	77.9 (49.5, 150.9)

Ejection fraction (%), mean ± SD	51 ± 9

Euroscore (%), mean ± SD	3.5 ± 3.2

Number of grafts, *n* (%)	
1	7 (5.4)
2	53 (40.8)
3	64 (49.2)
4	6 (4.6)

ACC^b^ (sec), mean ± SD	71.2 ± 34.2

CPB^c^ (sec), mean ± SD	100.5 ± 35.3

^a^SD: standard deviation.

^b^ACC: aortic cross-clumping.

^c^CPB: cardiopulmonary bypass.

**Table 2:** Plasma osteopontin levels in study population.

Characteristic	Patients, *n* (%)	Osteopontin, ng/mL Median (interquartile range)	*p* value
Gender			
Female	14 (10.7)	110.8 (59.4, 200.0)	0.103
Male	117 (89.3)	76.9 (48.2, 149.0)	

Diabetes			
Yes	50 (38.5)	83.8 (52.0, 160.0)	0.590
No		77.1 (49.4, 149.0)	

Hyperlipidemia			
Yes	113 (86.9)	80.1 (50.5, 150.9)	0.928
No		72.5 (54.9, 149.0)	

Hypertension			
Yes	121 (93.1)	80.1 (50.5, 149.0)	0.452
No		76.9 (54.9, 221.0)	

Smoking			
Yes	70 (53.8)	75.0 (45.5, 134.0)	0.071
No		88.5 (54.9, 167.0)	

Family history of coronary disease			
Yes	62 (48.1)	81.3 (52.0, 160.0)	0.607
No		76.9 (48.9, 134.0)	
